# Author Correction: Computer keyboard interaction as an indicator of early Parkinson’s disease

**DOI:** 10.1038/s41598-018-32121-x

**Published:** 2018-10-16

**Authors:** L. Giancardo, A. Sánchez-Ferro, T. Arroyo-Gallego, I. Butterworth, C. S. Mendoza, P. Montero, M. Matarazzo, J. A. Obeso, M. L. Gray, R. San José Estépar

**Affiliations:** 1 Madrid-MIT M+ Visión Consortium, Research Laboratory of Electronics, Massachusetts Institute of Technology, Cambridge, MA USA; 2 HM Hospitales - Centro Integral en Neurociencias HM CINAC, Móstoles, Madrid Spain; 3 CEU San Pablo University, Campus de Moncloa, Calle Julián Romea, 18, 28003 Madrid, Spain; 4 Centro de Investigación Biomédica en Red, Enfermedades Neurodegenerativas (CIBERNED), Madrid, Spain; 5 Instituto de Investigación Hospital 12 de Octubre (i+ 12), Madrid, Spain; 6 Universidad Politécnica de Madrid, Madrid, Spain; 7 Movement disorders unit, Hospital Clinico San Carlos, Madrid, Spain; 8 The Institute of Medical Engineering and Science, Massachusetts Institute of Technology, Cambridge, MA USA; 9 Brigham and Women’s Hospital, Harvard Medical School, Boston, MA USA

Correction to: *Scientific Reports* 10.1038/srep34468, published online 05 October 2016

This Article contains errors.

We were alerted to a potential error in one of our analyses. We then reviewed the analysis and found a mistake in the Receiver Operating Characteristic (ROC) curve computation for one of the finger tapping test (the alternating finger tapping test). This error was due to automatic conversion of the alternating finger tapping missing data to a value of 0 by the functions implemented in the “Scikit-learn” Python library^1^ used at the time of writing the paper (v. 0.13.1). The most recent versions fixed this problem.

We updated our “Scikit-learn” Python library and re-ran our statistical analysis. We then confirmed our results using the pROC package implemented in R^2^. We find that the Area under the ROC curve (AUC) for the alternative finger tapping test is 0.83, and not 0.75 reported in the published Article.

As a result, in the Abstract,

“The performance was comparable or better than two other quantitative motor performance tests used clinically: alternating finger tapping (AUC = 0.75) and single key tapping (AUC = 0.61).”

should read:

“The performance was comparable or better than two other quantitative motor performance tests used clinically: alternating finger tapping (AUC = 0.83) and single key tapping (AUC = 0.61).”

In the legend of Figure 3,

“The nQi score shows the best performance in comparison with alternating finger tapping (p < 0.001) and single key tapping (p < 0.001).”

should read:

“The nQi score shows comparable performance in to alternating finger tapping (p = 0.699) and superior to single key tapping (p = 0.01).”

and

“In our cohort, the former showed better performance than the latter (p = 0.008).”

should read:

“In our cohort, the former showed better performance than the latter (p = 0.004).”

The correct Figure 3 and accompanying legend, with correctly computed AUCs, appears below as Figure 1.

**Figure 1:**
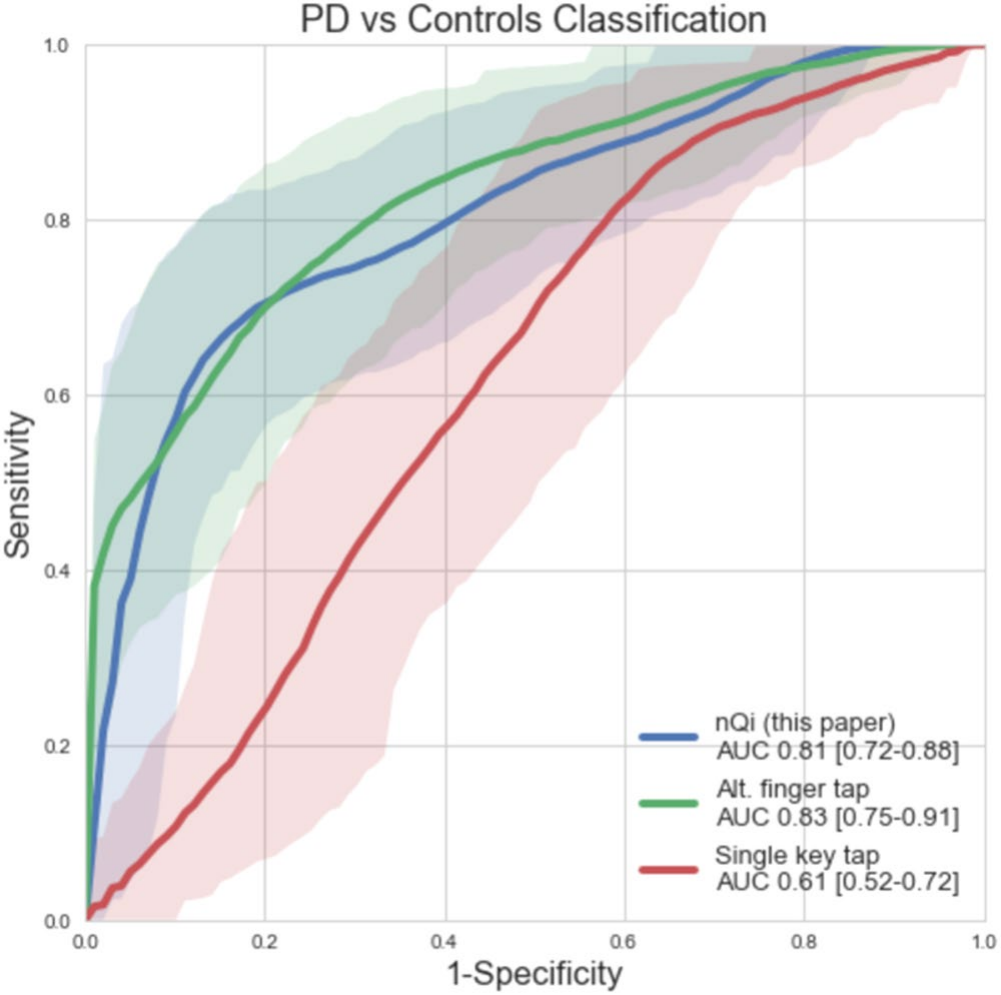
Comparison of receivers operating characteristic (ROC) curves showing the classification performance of nQi (main contribution of this paper), alternating finger tapping and single key tapping on the combined dataset of 42 PD subjects and 43 controls. The shadowed areas represent the 95% confidence intervals. In the legend, the area under the ROC curve (AUC) and the 95% confidence intervals and are shown (see Table 1 for more details). The nQi score shows comparable performance in to alternating finger tapping (p = 0.699) and superior to single key tapping (p = 0.01). Alternating finger tapping and single key tapping are two quantitative measurements commonly used to evaluate motor impairment in PD studies. In our cohort, the former showed better performance than the latter (p = 0.004). The p-values have been computed with the DeLong’s test for correlated ROC curves, which test the null hypothesis that the AUCs of two ROC curves are statistically the same.

These changes do not alter the conclusions of the Article.
