# Supplementary material for: Computer keyboard interaction as an indicator of early Parkinson’s disease
Source: Sci Rep. 2016 Oct 5;6:34468. doi: 10.1038/srep34468 (PMC5050498; doi:10.1038/srep34468)
Supplement: Supplementary Information [file srep34468-s1.pdf]

Supplementary Material for “Computer keyboard interaction as an indicator of early Parkinson’s disease”

L. Giancardo[1,†,*], A. Sánchez-Ferro[1-5,†], T. Arroyo-Gallego[1,6], I. Butterworth[1], C. S. Mendoza[1], P. Montero[7], M. Matarazzo[2-5], J. A. Obeso[2-4], M. L. Gray[1,8], and R. San José Estépar[9]

[1]Madrid-MIT M+Visión Consortium, Research Laboratory of Electronics, Massachusetts Institute of Technology, Cambridge, MA, USA
[2]HM Hospitales - Centro Integral en Neurociencias HM CINAC, Móstoles, Madrid, Spain
[3]CEU San Pablo University, Campus de Moncloa, Calle Julián Romea, 18, 28003 Madrid, Spain
[4]Centro de Investigación Biomédica en Red, Enfermedades Neurodegenerativas (CIBERNED), Madrid, Spain
[5]Instituto de Investigación Hospital 12 de Octubre (i+12), Madrid, Spain
[6]Universidad Politécnica de Madrid, Spain
[7]Movement disorders unit, Hospital Clinico San Carlos, Madrid, Spain
[8]The Institute of Medical Engineering and Science, Massachusetts Institute of Technology, Cambridge, MA, USA
[9]Brigham and Women’s Hospital, Harvard Medical School, Boston, MA, USA
[†]L.G. and A.S.F. have contributed equally to this work
[*]gianca@mit.edu

ABSTRACT

Parkinson’s disease (PD) is a slowly progressing neurodegenerative disease with early manifestation of motor signs. Objective measurements of motor signs are of vital importance for diagnosing, monitoring and developing disease modifying therapies, particularly for the early stages of the disease when putative neuroprotective treatments could stop neurodegeneration. Current medical practice has limited tools to routinely monitor PD motor signs with enough frequency and without undue burden for patients and the healthcare system. In this paper, we present data indicating that the routine interaction with computer keyboards can be used to detect motor signs in the early stages of PD. We explore a solution that measures the key hold times (the time required to press and release a key) during the normal use of a computer without any change in hardware and converts it to a PD motor index. This is achieved by the automatic discovery of patterns in the time series of key hold times using an ensemble regression algorithm. This new approach discriminated early PD groups from controls with an AUC=0.81 (n=42/43;mean age=59.0/60.1;women=43%/60%;PD/controls). The performance was comparable or better than two other quantitative motor performance tests used clinically: alternating finger tapping (AUC=0.75) and single key tapping (AUC=0.61).

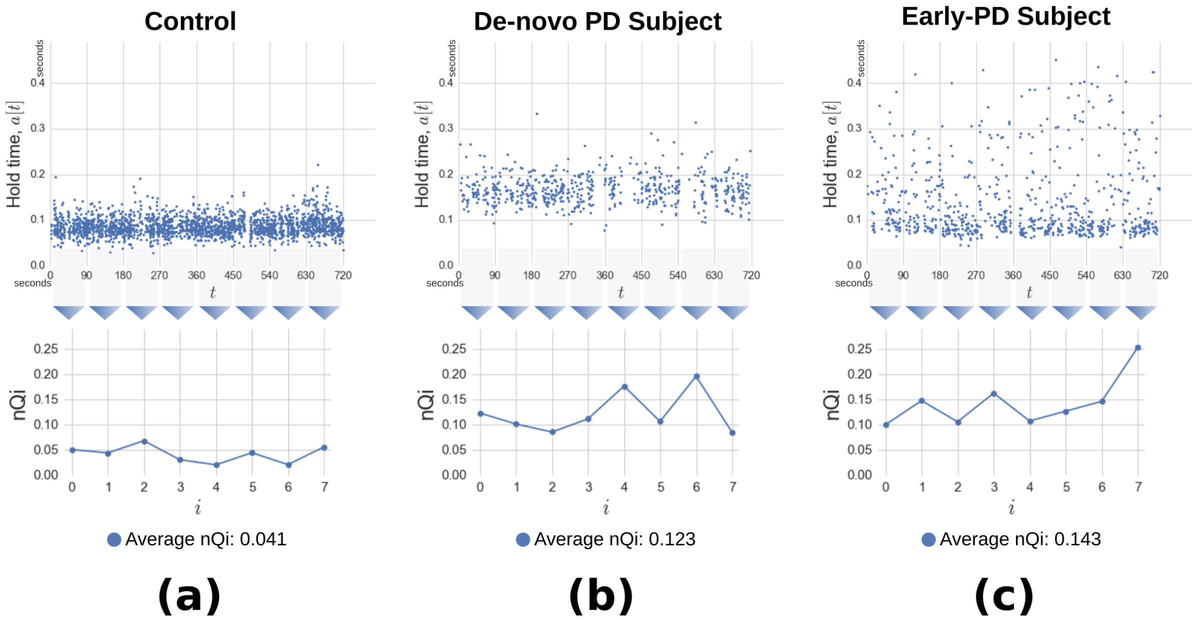

Figure S.1. Representative examples of the hold time (HT) time series and derived nQi scores. The HTs were generated from a typing task during which the subject transcribed a folk tale using a standard word processing program. (a) Control subject; (b) De-novo PD subject recently diagnosed with PD and not on any PD medication; (c) Early PD: a subject with PD, who is normally medicated, but has been without medication for 18 hrs prior to testing. The HT series are noticeably more variable in subjects with PD. The distribution and variability features of the HT provide a local nQi metric (as illustrated in Fig. 1); for the three subjects shown here, the average nQi(s) are 0.041, 0.123 and 0.143.

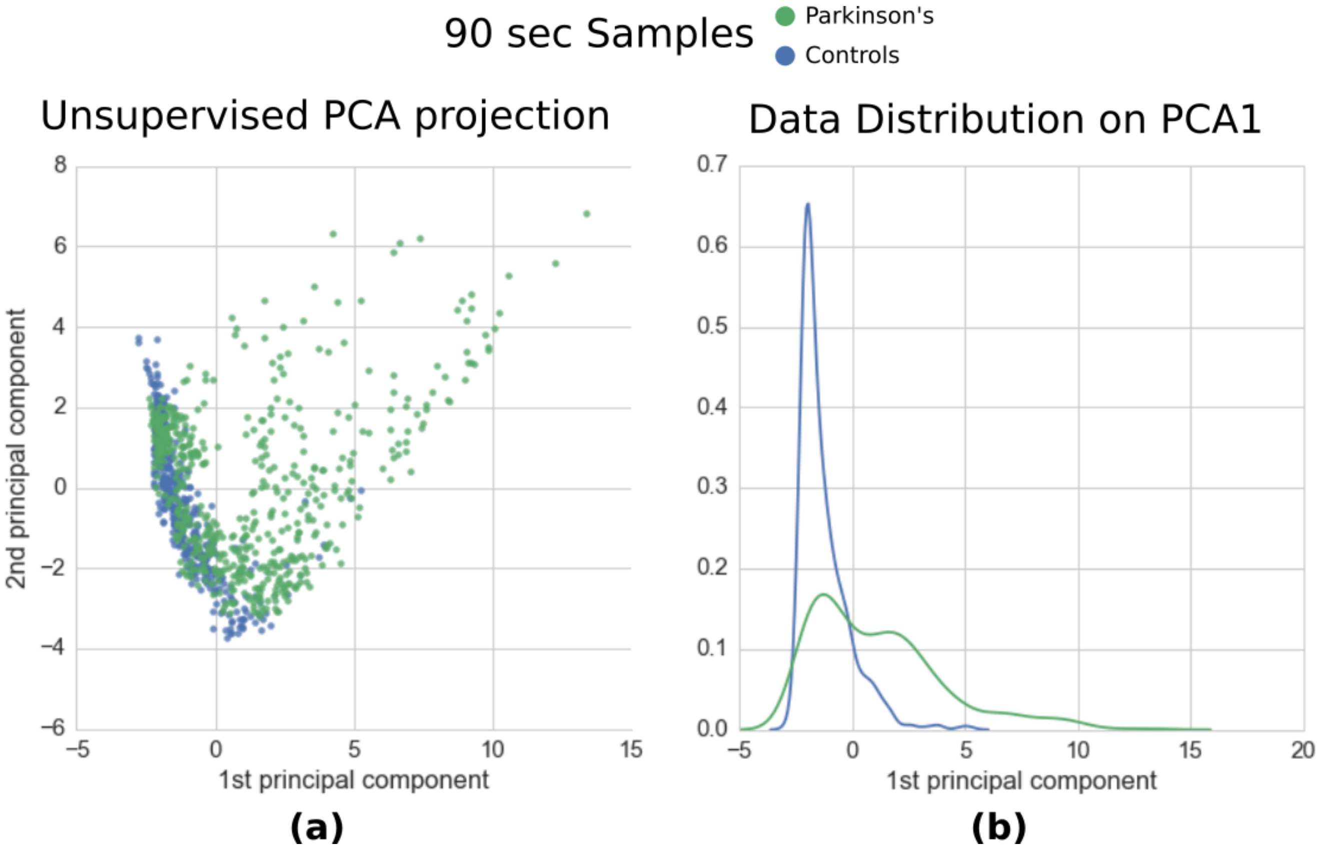

Figure S.2. Unsupervised Principal Component Analysis (PCA) on all 1088 feature vectors x_i of the combined dataset containing 43 controls, 44 PDs. (a) shows the projection of the vectors on the first two PCA components. It can be seen that only the samples coming from the Parkinson's group tend to go toward higher values in the 1st principal component and lower values in the 2nd principal component of PCA space, while the samples from controls appear much less disperse. (b) confirms this observation by showing the two distributions on the first PCA component only. Both PD and control group show a multimodal behavior as confirmed by the Hartigans' dip test. The hypotheses that the two groups follow Gaussian distributions is discarded by the Mardia's Multivariate Normality Test ($p<0.001$).

Control (n=43) / PD (n=42)

	coef	std err	z	P>|z|	[95.0% Conf.	Int.]
intercept	-2.5104	2.260	-1.111	0.267	-6.941	1.920
sex	0.6422	0.526	1.222	0.222	-0.388	1.672
age	0.0055	0.029	0.189	0.850	-0.051	0.062
typingSpeed	0.0005	0.006	0.079	0.937	-0.012	0.013
educ	0.0135	0.061	0.221	0.825	-0.106	0.133
nqScore	18.9401	5.799	3.266	0.001	7.575	30.306

(a)

Control (n=43) / De-novo PD (n=24)

	coef	std err	z	P>|z|	[95.0% Conf.	Int.]
intercept	0.4278	3.233	0.132	0.895	-5.909	6.765
sex	0.3802	0.739	0.515	0.607	-1.068	1.828
age	-0.0621	0.047	-1.320	0.187	-0.154	0.030
typingSpeed	-0.0080	0.009	-0.928	0.354	-0.025	0.009
educ	0.0665	0.087	0.769	0.442	-0.103	0.236
nqScore	21.4065	7.100	3.015	0.003	7.491	35.322

(b)

Control (n=43) / Early PD (n=18)

	coef	std err	z	P>|z|	[95.0% Conf.	Int.]
intercept	-2.6705	2.578	-1.036	0.300	-7.724	2.383
sex	0.5531	0.600	0.922	0.357	-0.623	1.729
age	0.0107	0.033	0.329	0.742	-0.053	0.074
typingSpeed	-0.0001	0.007	-0.017	0.987	-0.014	0.014
educ	0.0160	0.067	0.240	0.810	-0.115	0.147
nqScore	11.5481	5.049	2.287	0.022	1.653	21.443

(c)

Figure S.3. Logistic regression tests using the subject status (PD or control) as dependent variable with the combined dataset and two patient subgroups. The following independent variables are used: sex, age, typing speed (typing skills), years of education and nQi (nqScore). In all models (a,b,c), nQi showed statistical relevance.

Typing Speed
Control (n=43) / PD (n=42)

	coef	std err	z	P>|z|	[95.0% Conf.	Int.]
intercept	1.9517	1.770	1.103	0.270	-1.517	5.421
sex	0.6577	0.463	1.422	0.155	-0.249	1.565
age	-0.0295	0.025	-1.169	0.242	-0.079	0.020
educ	0.0210	0.056	0.372	0.710	-0.090	0.131
typingSpeed	-0.0081	0.005	-1.497	0.135	-0.019	0.003

(a)

Alternated Finger Tapping
Control (n=37) / PD (n=39)

	coef	std err	z	P>|z|	[95.0% Conf.	Int.]
intercept	12.8277	3.588	3.575	0.000	5.794	19.861
sex	0.0820	0.616	0.133	0.894	-1.126	1.290
age	-0.0864	0.034	-2.522	0.012	-0.154	-0.019
educ	0.0070	0.064	0.110	0.912	-0.118	0.133
afTap	-0.0699	0.016	-4.259	0.000	-0.102	-0.038

(b)

Single Key Tapping
Control (n=43) / PD (n=42)

	coef	std err	z	P>|z|	[95.0% Conf.	Int.]
intercept	5.5694	2.827	1.970	0.049	0.028	11.111
sex	0.9384	0.479	1.957	0.050	-0.001	1.878
age	-0.0253	0.023	-1.077	0.281	-0.071	0.021
educ	-0.0115	0.050	-0.230	0.818	-0.110	0.087
sTap	-0.0261	0.012	-2.113	0.035	-0.050	-0.002

(c)

Figure S.4. Logistic regression tests using the subject status (PD or control) as dependent variable and the following independent variables: sex, age, years of education and the metric to be tested. (a) Testing for group discrimination of typing speed; (b) testing group discrimination of alternated finger tapping. This test was introduced with the study on-going, because of this 5 PD subjects and 4 controls could only be measured with the single key tapping test and our typing test; (c) testing group discrimination of single key tapping.

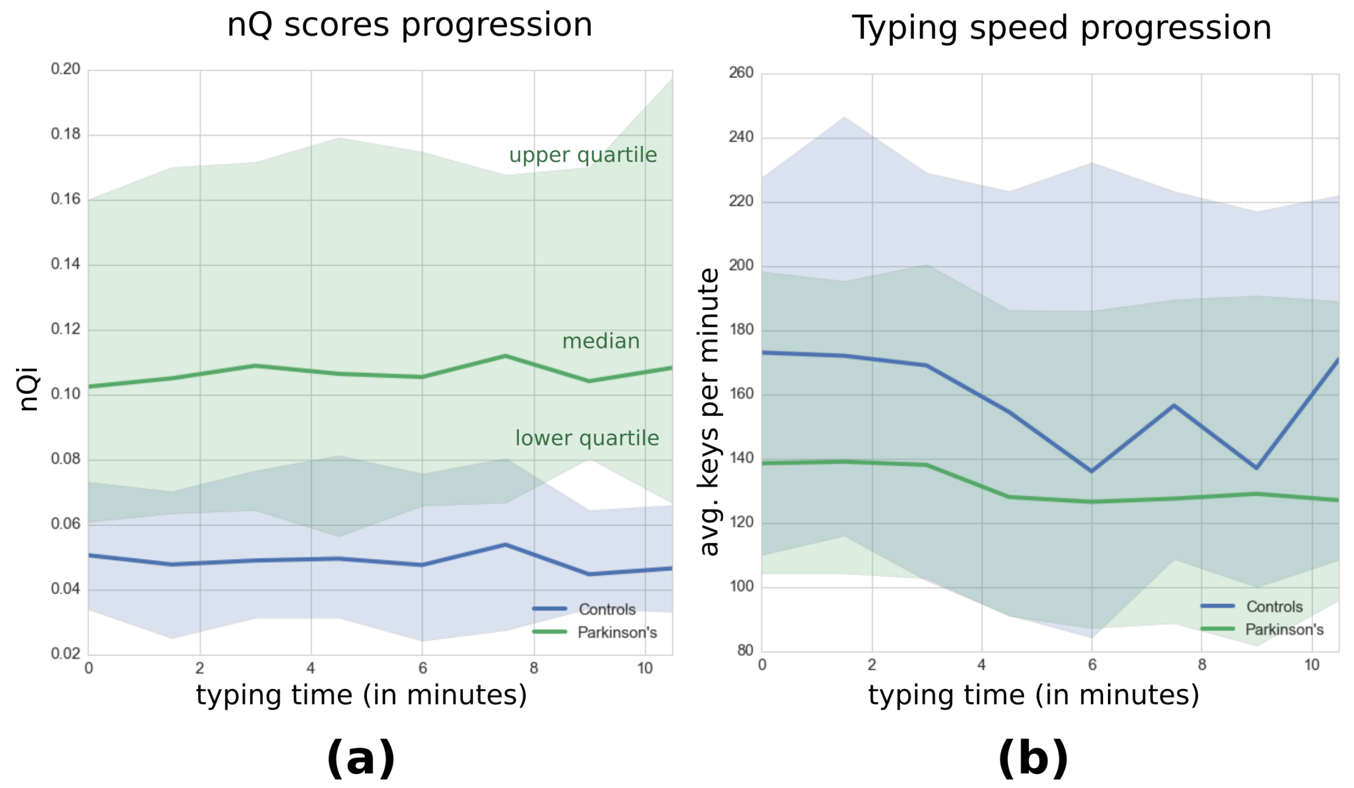

Figure S.5. Group level comparison of typing progression from the combined dataset (42 PD and 43 controls). The solid lines represent the group medians and the shadows the upper/lower quartiles. Medians and quartiles are computed with 90 seconds non-overlapping temporal windows. No temporal smoothing was performed. (a) Group nQi scores during the typing task. The grouped nQi medians appear stable and they are able discriminate PD from groups also assuming independent 90 seconds windows (AUC 0.79, 0.76-0.82 95% CI). (b) Typing speed, the main measure of typing skills, using the same data employed to compute the nQi scores shown on the left. Typing speed alone is very poor at distinguishing the groups also assuming independent 90 seconds windows (AUC 0.58, 0.54-0.62 95% CI).

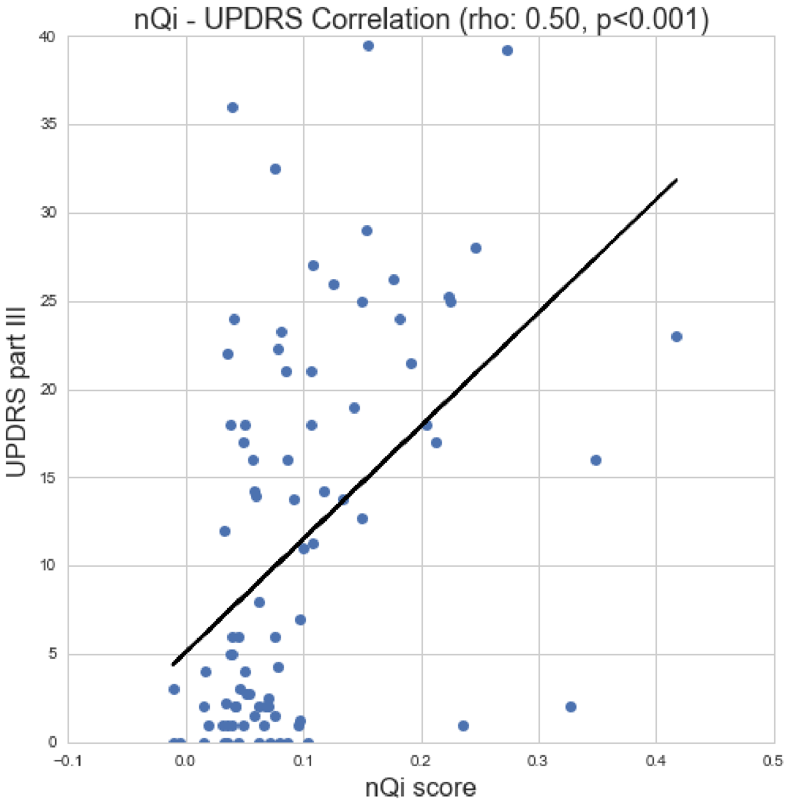

Figure S.6. Correlation between the clinical scores for UPDRS-III and nQi on the combined dataset of 42 PD subjects and 43 controls. nQi showed a moderate correlation with UPDRS-III (Spearman rho=0.50; $p<0.001$). Note that the dataset used does not represent the whole spectrum of PD, therefore the correlation found should be taken with care.

Subject ID	Group	Avg. UPDRS-III (0-108)	Avg. ATF	Avg. SKT	Avg. nQi	Typing Speed (# of keys per min)
11	PD	14.25	NaN	162.25	0.118	189.4
60	CNT	2	NaN	162.25	0.070	60.5
67	PD	25.25	NaN	133.75	0.223	54.3
68	CNT	6	NaN	159	0.075	71.8
70	PD	26.25	NaN	113.5	0.176	39.6
71	PD	13.75	132.5	153	0.133	106.3
72	PD	17	NaN	146.75	0.212	79.1
73	PD	23.25	99.25	164.25	0.080	115.5
74	PD	11.25	NaN	175.5	0.108	186.1
75	PD	12.75	79.25	158.75	0.149	176.1
76	CNT	0	136.75	177	0.086	249.7
77	CNT	2.25	NaN	150	0.035	79.7
78	PD	39.25	49.5	191.75	0.273	85.0
79	CNT	1.25	148.75	173.75	0.097	82.5
80	PD	39.5	92	125.5	0.155	70.7
81	CNT	4.25	NaN	158	0.078	57.5
82	PD	32.5	96	196.75	0.075	33.5
83	CNT	2.75	152	164.5	0.052	110.7
84	PD	22.25	97.75	156.25	0.078	99.9
85	CNT	2.5	107.75	159.5	0.070	104.1
86	PD	14.25	114.75	133.25	0.058	119.8
87	CNT	2.75	118.25	140.5	0.054	188.0
88	PD	13.75	95	157	0.091	105.2
89	CNT	0	144.25	164.5	0.080	149.3
92	PD	21.5	101.25	145	0.191	81.0
93	PD	21	116	190.75	0.107	80.9
94	CNT	4	142	157.25	0.050	85.5
95	CNT	1.5	213	181.75	0.075	188.8
97	PD	25	92	188.75	0.150	97.0
98	PD	29	87	176.75	0.153	59.9

Table S.1. Data summary for the Early-PD dataset. UPDRS-III: Unified Parkinson's Rating Scale part III; ATF: alternated finger tapping (when NaN the information is not available); SKT: single key tapping.

Subject ID	Group	Avg. UPDRS-III (0-108)	Avg. ATF	Avg. SKT	Avg. nQi	Typing Speed (# of keys per min)
1000	PD	27	79	184.5	0.107	56.9
1001	PD	16	96.5	189	0.056	118.0
1002	CNT	5	140	158	0.040	119.0
1004	PD	22	83.5	191.5	0.035	74.3
1005	PD	17	68	150	0.048	75.0
1006	PD	18	93	140.5	0.038	112.9
1008	PD	18	130	169	0.051	182.1
1009	PD	19	82.5	176	0.143	64.7
1010	CNT	2	135	197.5	0.042	172.3
1011	CNT	1	120	174.5	0.035	97.1
1012	CNT	4	172.5	190.5	0.016	128.4
1013	CNT	1	126	177.5	0.096	126.1
1014	PD	24	96	170	0.182	100.2
1015	CNT	2	117.5	201.5	0.041	65.8
1016	CNT	6	167.5	195.5	0.045	174.6
1017	PD	23	61	150.5	0.417	30.7
1019	PD	7	94	164.5	0.097	121.5
1020	PD	18	119	160	0.106	90.6
1021	CNT	0	96.5	163.5	0.045	78.7
1022	CNT	2	142	179	0.067	131.0
1023	PD	11	117.5	144	0.100	138.0
1024	PD	14	122.5	170	0.059	153.1
1025	PD	18	131.5	174	0.205	49.6
1028	PD	24	110.5	200.5	0.041	119.6
1029	CNT	2	161.5	172.5	0.062	61.2
1030	CNT	3	152.5	190.5	0.046	163.1
1031	CNT	1	121	179	0.031	178.3
1032	CNT	6	149.5	182	0.040	101.4
1033	CNT	1	111	169	0.040	84.7
1034	PD	36	58.5	174	0.039	149.7
1035	CNT	1	120.5	195.5	0.019	65.3
1037	PD	21	65.5	156.5	0.085	81.2
1039	CNT	0	141.5	167	-0.011	170.7
1041	PD	16	74	186	0.349	40.4
1042	CNT	0	145	175.5	0.035	234.9
1043	CNT	3	101.5	191	-0.011	68.1
1045	CNT	1	131	174.5	0.048	137.6
1047	PD	25	106.5	137	0.225	35.8
1049	CNT	1	84	123.5	0.066	114.7
1050	CNT	5	129.5	170.5	0.038	51.2
1051	CNT	1	89	138.5	0.235	26.4
1052	PD	16	128	213	0.086	152.8
1053	CNT	0	90	178.5	0.032	42.5
1055	CNT	0	126.5	167.5	0.104	21.8
1056	CNT	0	162	188	0.062	257.2
1057	CNT	0	66.5	157.5	0.072	23.8
1059	PD	8	100.5	162.5	0.062	91.4
1061	CNT	0	120.5	157	0.015	109.0
1062	CNT	2	115.5	179	0.326	140.5
1063	CNT	0	110	170	-0.006	109.8
1064	PD	28	75.5	140	0.247	105.3
1066	PD	12	118	170.5	0.033	140.3
1068	PD	26	65.5	98	0.126	48.8
1070	CNT	2	126	154.5	0.015	54.9

Table S.2. Data summary for the Early-PD dataset. UPDRS-III: Unified Parkinson's Rating Scale part III; ATF: alternated finger tapping (when NaN the information is not available); SKT: single key tapping.
